# Rate of force development: physiological and methodological considerations

**DOI:** 10.1007/s00421-016-3346-6

**Published:** 2016-03-03

**Authors:** Nicola A. Maffiuletti, Per Aagaard, Anthony J. Blazevich, Jonathan Folland, Neale Tillin, Jacques Duchateau

**Affiliations:** Human Performance Lab, Schulthess Clinic, Lengghalde 6, 8008 Zurich, Switzerland; Department of Sports Science and Clinical Biomechanics, SDU Muscle Research Cluster (SMRC), University of Southern Denmark, Odense, Denmark; Centre for Exercise and Sports Science Research (CESSR), School of Medical and Health Sciences, Edith Cowan University, Joondalup, Australia; School of Sport, Exercise and Health Sciences, Loughborough University, Loughborough, UK; Department of Life Sciences, University of Roehampton, London, UK; Laboratory of Applied Biology, ULB Neurosciences Institute, Université Libre de Bruxelles (ULB), Brussels, Belgium

**Keywords:** Explosive strength, Ballistic contraction, Motor unit discharge rate, Musculotendinous stiffness, Strength training, Dynamometry

## Abstract

The evaluation of rate of force development during rapid contractions has recently become quite popular for characterising explosive strength of athletes, elderly individuals and patients. The main aims of this narrative review are to describe the neuromuscular determinants of rate of force development and to discuss various methodological considerations inherent to its evaluation for research and clinical purposes. Rate of force development (1) seems to be mainly determined by the capacity to produce maximal voluntary activation in the early phase of an explosive contraction (first 50–75 ms), particularly as a result of increased motor unit discharge rate; (2) can be improved by both explosive-type and heavy-resistance strength training in different subject populations, mainly through an improvement in rapid muscle activation; (3) is quite difficult to evaluate in a valid and reliable way. Therefore, we provide evidence-based practical recommendations for rational quantification of rate of force development in both laboratory and clinical settings.

## Introduction

Explosive strength is the ability to increase force or torque as quickly as possible during a rapid voluntary contraction realised from a low or resting level. Rate of force development (RFD), which is derived from the force- or torque-time curves recorded during explosive voluntary contractions (Aagaard et al. 2002a)—hereafter also referred to as rapid or ballistic actions—is increasingly evaluated to characterise explosive strength of athletes, elderly individuals and patients. This is mainly due to the facts that, as compared to pure maximal voluntary contraction (MVC) strength, RFD seems to be (1) better related to most performances of both sport-specific and functional daily tasks (see, e.g., Maffiuletti et al. 2010; Tillin et al. 2013a), (2) more sensitive to detect acute and chronic changes in neuromuscular function (see, e.g., Angelozzi et al. 2012; Crameri et al. 2007; Jenkins et al. 2014b; Penailillo et al. 2015) and (3) potentially governed by different physiological mechanisms (see, e.g., Andersen and Aagaard 2006; Van Cutsem et al. 1998). The ability to properly quantify and interpret RFD obtained during voluntary isometric contractions is therefore extremely important not only for researchers in the field of human and exercise physiology, but also for practitioners in the fields of physical training and rehabilitation.

The aim of this narrative review is to provide the main physiological and methodological considerations for ensuring accurate assessment and interpretation of RFD in research and clinical settings. A better knowledge of these critical aspects is helpful for designing interventions not only to increase explosive force production in athletes but also to improve physical function and reduce injury and fall risk in elderly and patient populations.

## Physiological considerations: the underlying mechanisms

The purpose of this section is to review the main neuromuscular mechanisms underlying explosive strength and to discuss their potential contribution to RFD. This section examines successively the neural and muscular determinants of RFD and describes the neuromuscular adaptations to training that influence RFD.

### Neural determinants

#### Motor unit (MU) recruitment and discharge rate

Despite some technical limitations (Farina et al. 2010), muscle activation is often assessed non-invasively by the use of surface electromyography. Using this approach, contrasting electromyogram (EMG) patterns are observed when comparing “slow” and “rapid” contractions. Whereas a monotonic augmentation in EMG activity is classically observed during a contraction realized with a slow and progressive (ramp-like) increase in force, a rapid/ballistic contraction is characterised by a highly synchronised burst of activity at the onset of the action (Bawa and Calancie 1983; Desmedt and Godaux 1977; Van Cutsem et al. 1998). The magnitude of the activation, and hence the force produced by a muscle, depends on the number of MUs activated (MU recruitment) and the rates at which motor neurones discharge action potentials (rate coding). The recruitment order of MUs during rapid contractions is similar to that observed during slower contractions and follows the “size principle” (i.e., low threshold units are recruited before larger ones) (see Duchateau and Enoka 2011). However, the relative contributions of recruitment and discharge rate modulation to the force exerted by a muscle vary with contraction speed. Slow contractions are characterised by a progressive activation of MUs to an upper limit of recruitment that reaches ~80–90 % of the maximum force in most limb muscles (De Luca et al. 1982; Kukulka and Clamann 1981; Van Cutsem et al. 1997). In contrast, MUs are recruited at much lower forces (i.e., lower recruitment thresholds) during rapid actions. For example, most MUs in the tibialis anterior are recruited during a ballistic contraction when the force is only 1/3 of maximum (Desmedt and Godaux 1977). Furthermore, the reduction in recruitment threshold of MUs with increased contraction speed is more notable for slow-contracting (e.g., soleus) than for fast-contracting muscles (e.g., masseter) (Desmedt and Godaux 1978). As a consequence, the increase in muscle force beyond the upper limit of MU recruitment is entirely due to an increased discharge rate. In most muscles, the maximal rate at which MUs discharge action potentials during sustained, high-force isometric contractions is 30–60 Hz (Duchateau and Enoka 2011). In contrast, the instantaneous MU discharge rates at the onset of a rapid contraction often reach values of 60–120 Hz in untrained subjects (Desmedt and Godaux 1977) and above 200 Hz in trained individuals. During such rapid actions, the activated MUs discharge only a few times (~1–6) (Van Cutsem and Duchateau 2005; Van Cutsem et al. 1998). Contrary to slow contractions, during which the discharge rate of MUs increases progressively, rapid contractions are characterised by a high initial discharge rate at the onset of activation that declines progressively with successive discharges (Desmedt and Godaux 1977; Klass et al. 2008; Van Cutsem et al. 1998). These observations underscore the speed-related difference in MU activation pattern.

#### Association between muscle activation and RFD

**Fig. 1:**
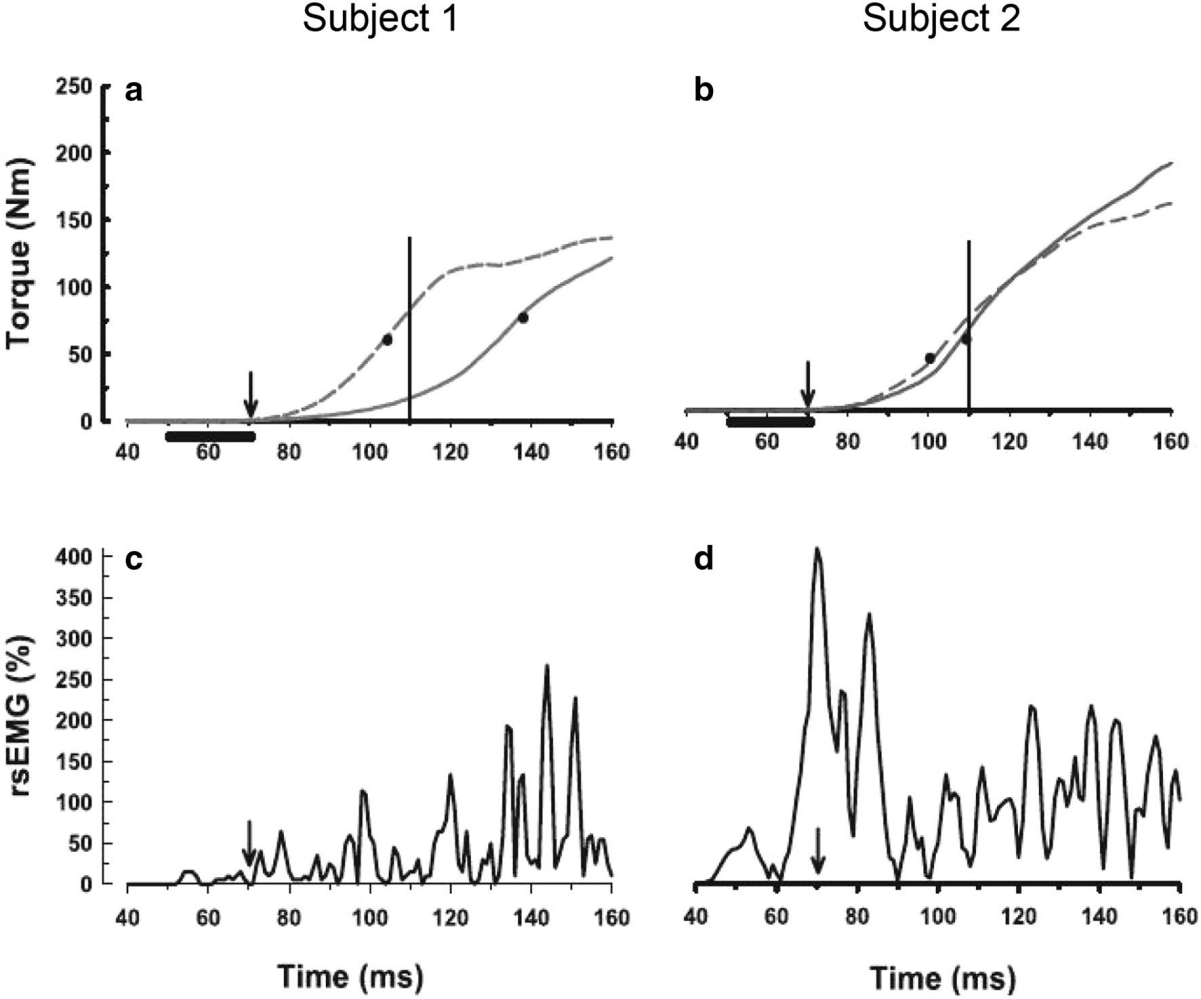
Illustration of the early phase of the torque- and EMG-time *curves* of the knee extensor muscles in two subjects during an explosive voluntary isometric contraction (*continuous line*) and in response to electrical stimulation (8 pulses at 300 Hz; *discontinuous line*). The rate of torque development during the two types of contraction is similar for subject 2 (**b**), whereas it is substantially smaller during voluntary activation for subject 1 (**a**). Interestingly, EMG activity (expressed as % of EMG during MVC) of the vastus lateralis is much greater at the onset of muscle activation in subject 2 (**d**) compared to subject 1 (**c**). These data indicate that the rate of torque development is mainly limited by muscle activation (neural factors) in subject 1 whereas it is more likely constrained by muscular factors in subject 2. *Arrows* and *vertical lines* indicate, respectively, the onset of torque development and the force attained 40 ms after the onset of the contraction. Figure reproduced with permission from de Ruiter et al. (2004)

The relative contribution of neural and muscular factors to the performance of a rapid contraction has been investigated by comparing RFD in contractions induced by voluntary activation and electrical stimulation. Using such an approach, several studies have shown that muscle activation, as assessed by surface EMG, is a major factor influencing the expression of RFD in vivo (Blazevich et al. 2009; de Ruiter et al. 2004; Del Balso and Cafarelli 2007; Folland et al. 2014; Klass et al. 2008). For example, and despite a great variability among subjects (see Fig. 1), the force attained 40 ms after the onset of a rapid voluntary knee extension was less (−60 %) than for an electrically induced tetanic contraction (de Ruiter et al. 2004). Furthermore, voluntary force, expressed relative to the force produced by electrical stimulation at the same time point, was positively associated (*r*^2^ = 0.76) with the surface EMG of the quadriceps before the onset of force development but not with that induced by electrical stimulation. These results indicate that the ability to produce force rapidly depends predominantly on the increase of muscle activation at the onset of the contraction and less on the speed-related properties of the muscle. Such a conclusion is supported indirectly by the work of Andersen and Aagaard (2006), who observed a moderate association (*r*^2^ = 0.36) between voluntary RFD measured in the first 40 ms of a rapid contraction of the knee extensors and electrically evoked twitch contractile properties. This suggests that mechanisms other than intrinsic muscle properties explain most of the variance of voluntary RFD.

Because the relative contribution of neural and contractile factors may vary during the early and late phases of rising force during a rapid contraction, Folland et al. (2014) examined their relative contribution throughout the rising phase of the force–time curve by recording surface EMG activity in the knee extensors. On the basis of multiple linear regressions, the contribution of neural (volitional EMG activity) and contractile (force responses to evoked twitch and octet (train of 8 pulses at 300 Hz) contractions) determinants of voluntary explosive force attained at different time points was analysed. A major finding was that the primary determinant of the voluntary RFD changed throughout the rising phase of the contraction. The results indicated that agonist EMG activity was an important contributor to the explained variance in force throughout the entire 150 ms of rising muscle force, but more particularly in the initial phase (25–75 ms). In contrast, the evoked RFD evaluated from the octet (tetanic) contraction (reflecting intrinsic muscle contractile properties) was the primary determinant of the steeper phase of voluntary RFD (50–100 ms). These results are consistent with previous studies indicating a role for neural factors at the onset (<75 ms) of a rapid contraction (Aagaard et al. 2002a; de Ruiter et al. 2004; Del Balso and Cafarelli 2007; Klass et al. 2008; Van Cutsem et al. 1998). For contractions of longer duration (>75 ms), however, the voluntary RFD becomes more strongly influenced by the speed-related properties of the muscle and MVC force per se (Andersen and Aagaard 2006; Folland et al. 2014).

#### Association between maximal MU discharge rate and RFD

As described in the preceding paragraph, the neural input to the muscle is of functional importance for muscle performance at the onset of rapid contractions. In that context, parallel changes in RFD and discharge characteristics of MUs during ballistic isometric ankle dorsiflexions have been reported when the initial condition was modified. For example, the average discharge rate for the first three interspike intervals in tibialis anterior was reduced by 22 % and RFD by 16 % when ballistic contractions were superimposed on a submaximal isometric contraction (Van Cutsem and Duchateau 2005). Such concurrent changes have also been observed with long-term adaptations such as with ageing (Klass et al. 2008). In agreement with the reduced speed-related capacity associated with ageing, older adults (71–84 years) displayed a much slower RFD (−48 %) and MU discharge rate (−27 %) at the activation onset of ballistic ankle dorsiflexions compared with young subjects (~20 years). Furthermore, the number of doublet discharges (an index of motor neurone excitability) at brief interspike intervals (<5 ms) was reduced by about half in older adults (4.6 vs. 8.4 % of total number of MUs).

In an attempt to document in humans, the results of Buller and Lewis (1965) and de Haan (1998) showing the importance of MU activation rate on RFD in animal muscles (cat and rat), a simulation study was performed by Duchateau and Baudry (2014). For that purpose, mechanical properties of MUs obtained using the spike-triggered averaging method recorded in the tibialis anterior (Van Cutsem et al. 1997) were inserted into the model developed by Fuglevand et al. (1993). The results indicated that an increase in discharge rate up to 100–200 Hz substantially augmented the RFD of all units within the MU pool. As expected, further increases in discharge rate had less influence except for the faster units, thus reflecting a difference in speed-related properties between low- and high-threshold MUs (Kernell 2006).

Together these experimental and simulated studies underscore the critical role of maximal MU discharge rate on the ability to rapidly develop force at the onset of a ballistic voluntary contraction.

#### Limiting mechanisms of MU discharge rate

As for MVCs (Allen et al. 1995), there is a great inter-individual variability in the magnitude of muscle activation during rapid contractions (de Ruiter et al. 2004; Folland et al. 2014; Klass et al. 2008). Such variability is greater during the early phase of the contraction (first 40–50 ms) meaning that neural factors contribute substantially to the between-subject variance (Folland et al. 2014). Potential mechanisms that may explain such a deficit in voluntary activation likely involve various loci within the nervous system (Duchateau and Enoka 2002).

Different supraspinal centres are involved in the initiation of a motor action as well as the acquisition of a new motor skill (Doyon and Benali 2005). Presumably, part of the deficit in muscle activation during a rapid contraction is due to the inability to generate sufficient volitional drive in a brief period of time. In addition to a suboptimal output from the primary motor cortex, part of this submaximal performance can be due to an ineffective activation pattern of the agonist and antagonist/synergist muscles involved in the task (Duchateau and Enoka 2002; Geertsen et al. 2008, 2011). It is, however, not currently possible to precisely determine the reasons why volitional drive from the brain is insufficient to maximally activate muscles during rapid motor actions (Duchateau and Baudry 2014). Such differences in voluntary activation between individuals may reflect differences not only in their inherent capacity to produce rapid coordinated actions with a well-focused efferent drive to the involved agonist (and antagonist) muscles but also in their training status. Indeed, differences in muscle activation between subjects can be reduced quickly through learning, and even simple movements performed with muscles that only span a single joint can be improved substantially after a limited number of repetitions in a single practice session (Hinder et al. 2011; Jensen et al. 2005; Lee et al. 2010; Muellbacher et al. 2002; Rogasch et al. 2009). For example, Lee and colleagues (Lee et al. 2010) reported that multiple sets (each consisting of ten repetitions) of index finger abductions performed as fast as possible increased peak abduction acceleration by 64 and 93 % after 150 and 300 repetitions, respectively. The increase in acceleration was accompanied by an augmented corticospinal excitability of the first dorsal interosseous (43 and 63 % after 150 and 300 repetitions, respectively), as assessed by the size of the motor evoked potential induced by transcranial magnetic stimulation. In agreement with other experiments (Hinder et al. 2011; Lee et al. 2010; Muellbacher et al. 2002; Rogasch et al. 2009), this observation indicates that the motor cortex is implicated in motor learning and that the rapid improvement in such high-speed actions depends on short-term changes at the motor cortex level. This suggestion is consistent with the observation of an increased motor evoked potential in response to transcranial magnetic stimulation without change in motor evoked potential amplitude when the descending tract was stimulated at the cervico-medullary junction to bypass cortical centres (Muellbacher et al. 2001). Together, these studies indicate that small differences in the rate of acquisition processes in an unusual motor task likely contribute to the inter-individual variability observed during a rapid action.

It is well accepted that arousal can further influence the neural command sent to the muscles, thereby modulating physical performance (Jokela and Hanin 1999), muscle strength (Schmidt et al. 2009) and possibly RFD. Although little is currently known about the nature of these changes, recent animal studies have shown that monoaminergic input from the brainstem can change the gain of the MU pool to adjust their excitability to the requirements of the task (Heckman and Enoka 2012). Modulation can be provided by input from the noradrenergic system that is known to vary with arousal (Aston-Jones et al. 2000). Furthermore, changes in the activity of the serotonergic system appear to modulate the responsiveness of the motor neurones (Wei et al. 2014). In addition to the different proportion of slow and fast motor neurones between individuals and their respective intrinsic capacity to discharge at high rates (Desmedt and Godaux 1978), intra- and inter-individual variations in the level of these neuromodulatory inputs might influence the response of spinal motor neurones to the descending command. Furthermore, the observations that the number of doublet discharges decreases with ageing and the instantaneous MU discharge rate declines from the onset of muscle activation during rapid actions in untrained, and even more in aged individuals (Klass et al. 2008), suggest that intrinsic motor neurone properties and/or concurrent involvement of spinal inhibitory mechanisms may also modulate the level of muscle activation.

Despite some progress in recent years, more knowledge is needed to understand the inter-individual variability in the capacity to produce maximal voluntary activation during rapid muscle contractions.

### Muscular determinants

While rapid muscle activation is a critical determinant of RFD, muscular factors evidently also play a role. For example, the large inter-muscular (e.g., plantar flexors vs. knee extensors) and inter-individual differences in RFD cannot be explained completely on the basis of differences in muscle activation rates, and MVC force is strongly associated with late-phase RFD (Andersen and Aagaard 2006) so factors such as muscle size and architecture as well as others that influence muscle strength might also influence RFD.

#### Muscle fibre type composition

**Fig. 2:**
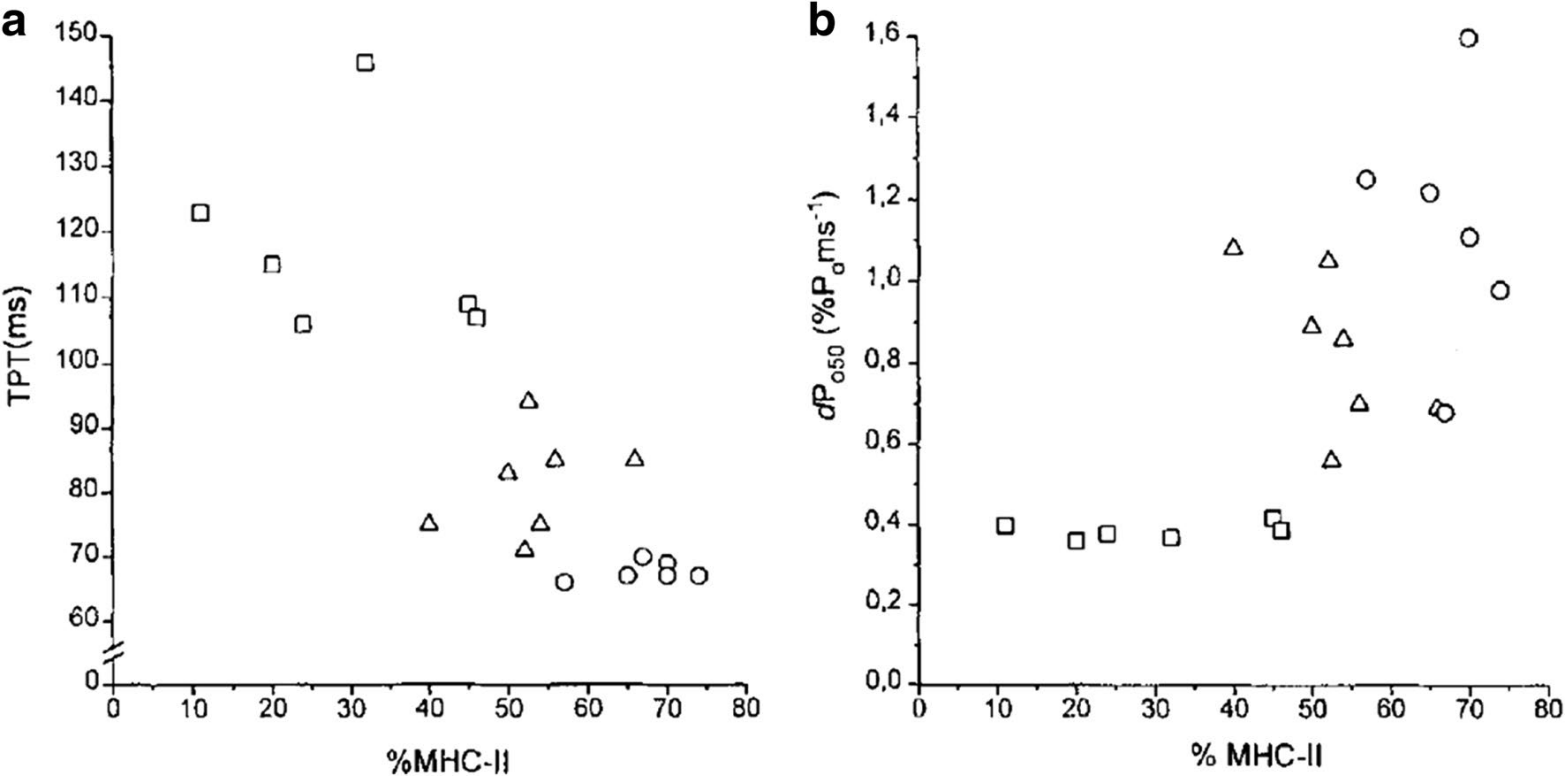
Distribution of myosin heavy chain (MHC)-II (IIA + IIB) isoforms in soleus (*squares*), vastus lateralis (*triangles*) and triceps brachii (*circles*) in relation to **a** twitch time to peak torque (TPT), and **b** maximal rate of rise of evoked torque at 50 Hz (dPo50). Between-muscle differences were strongly associated with differences in muscle fibre type, although the relationship with within-muscle variability was less pronounced. Figure reproduced with permission from Harridge et al. (1996)

Although caution is required when deducing the functional significance of differences in single fibre properties (see Enoka and Duchateau 2015), fibre type is often considered a major factor influencing muscular RFD based on the observation that the rate of tension development is faster in type II than type I fibres (Buchthal and Schmalbruch 1970; Harridge et al. 1996). This phenomenon is related to type II fibres having a greater total Ca^2+^ release per action potential (Baylor and Hollingworth 1988) and faster time constants of Ca^2+^ currents (e.g., Close 1972), as well as fast myosin, troponin and tropomyosin isoforms (Schiaffino and Reggiani 1996) and thus faster cross-bridge cycling rates (Bottinelli et al. 1996). There is a large variability in fibre type composition between various skeletal muscles. For example, vastus lateralis, vastus medialis and vastus intermedius contain ~50 % type I fibres (Johnson et al. 1973; Luden et al. 2008; Taylor et al. 1997) whilst soleus contains 75 % and gastrocnemius 45–75 % type I fibres (Dahmane et al. 2005; Edgerton et al. 1975; Johnson et al. 1973; Luden et al. 2008). Furthermore, very large inter-individual differences in human skeletal muscle fibre type composition also exist, for instance in the quadriceps femoris. Indeed, the percentage of type II fibres in vastus lateralis was found to vary between 25 and 80 % in a group of 21 randomly selected young untrained men, with no clear “average” fibre type percentage amongst the group (Andersen 2001). These differences in fibre type are qualitatively linked to inter-muscular differences in RFD. For example, Harridge et al. (1996) found that RFD (normalised to maximum force) elicited by 50-Hz electrical stimulation increased in the order plantar flexors < knee extensors < elbow extensors, which was consistent with the increase in type II myosin heavy chain percentages of soleus (30 %) < vastus lateralis (53 %) < triceps brachii (67 %) (Fig. 2; the same was observed for type II fibre percentages). Inter-individual differences in RFD measured in the same muscle group have also been correlated with fibre type in humans. Taylor et al. (1997) reported a moderate, albeit non-significant, correlation (*r*^2^ = 0.34) between vastus lateralis type II percentage and maximal voluntary knee extensor RFD, whilst Hvid et al. (2010) reported a significant correlation (*r*^2^ = 0.49) between vastus lateralis type II fibre area and knee extensor RFD measured to 50 ms in young men, although this was not observed in older men. Despite fibre type proportion having a substantial genetic basis (~50 %) (Simoneau and Bouchard 1995), it is also characterised by a high degree of adaptability (cf. Andersen and Aagaard 2000), and changes in fibre type have been linked to changes in RFD in the early rise in force (Hakkinen et al. 1985). These associations between fibre type and RFD measured in the early time phase of force rise are consistent with the moderate-to-strong correlations observed between RFD measured during twitch and voluntary contractions (Andersen and Aagaard 2006; Folland et al. 2014), given that twitch contractile properties are strongly influenced by muscle fibre type (e.g., Harridge et al. 1996). These data indicate that fibre type composition may be an important discriminating factor for inter-individual and inter-muscular differences in RFD measured particularly in the early force rise.

Nonetheless, a large portion of the explained variance in inter-individual or longitudinal changes in voluntary RFD in previous studies remained unaccounted for by myofibre properties (e.g., ~70 % in Hakkinen et al. 1985; ~60 % in Taylor et al. 1997; ~50 % in Hvid et al. 2010), indicating that other factors in the muscle–tendon unit and/or the nervous system must account for much of the variance.

#### Myofibrillar mechanisms

Prolonged or transient (e.g., milliseconds) (Abbate et al. 2002; Cheng et al. 2013) increases in action potential-stimulated Ca^2+^ release, and thus muscular force output, occur when higher frequency trains or short bursts (at contraction onset), respectively, propagate along the sarcolemma as input signals for muscle contraction. Changes in processes influencing this input–output relationship are thus likely to influence RFD (Wahr and Rall 1997). Exercise training, including 5 weeks of cycle sprint training in humans (Ortenblad et al. 2000), increases the total amount of sarcoplasmic reticulum in skeletal muscle, which can allow for a greater diffusion of excitatory potentials and a greater total number of voltage-sensitive dihydropyridine and Ca^2+^-release ryanodine receptors. Thus, the rate and magnitude of Ca^2+^ release can increase. Indeed, moderate-intensity exercise training has increased dihydropyridine content in both type I and II fibres (Ferreira et al. 2010; Saborido et al. 1995), and increases in exercise-induced upregulation of dihydropyridine gene expression are commonly observed in animal models (Manttiri et al. 2006; Anttila et al. 2006). These changes appear to be associated with an increase in Ca^2+^ release rate (e.g., measured as the AgNO_3_-induced Ca^2+^ rate) (Ortenblad et al. 2000) even when no change in ryanodine receptor density is observed. Subsequent research is required to determine whether such changes meaningfully impact RFD in vivo.

As evidenced by the effects of post-activation potentiation on RFD, increases in the sensitivity of the acto-myosin complex to Ca^2+^ might also influence RFD. Type II fibres have a lower Ca^2+^ sensitivity than type I fibres (Gardetto et al. 1989; Metzger and Moss 1990) leaving them particularly susceptible to stimuli that improve sensitivity (Grange et al. 1993). Repeated muscular contractions promote kinase-dependent phosphorylation of the myosin regulatory light chain that render the complex more sensitive to Ca^2+^, allowing a greater force production for a given Ca^2+^ release and thus an increase in the rate of force rise. In support of this notion, Vandenboom et al. (1993) found that increases in both twitch and tetanic maximal RFD in fast-twitch mouse extensor digitorum longus muscle elicited by a 20-s, 5-Hz electrical stimulation conditioning stimulus were strongly correlated with the increase in light chain phosphate content (*r*^2^ = 0.94 and 0.92, respectively), and increases in RFD after similar electrical stimulation protocols (and presumably similar increases in phosphorylation) have been observed in humans (Baudry and Duchateau 2007; Hamada et al. 2000).

Nonetheless, notwithstanding the lack of substantial evidence, changes in Ca^2+^ sensitivity may not have a particularly strong influence on RFD in many circumstances. For example, despite Ca^2+^ sensitivity often being shown to be greater in type I than II fibres (Hvid et al. 2011; Malisoux et al. 2006; Fitts and Widrick 1996), this cannot explain the finding of a greater RFD in muscles (or individuals) with a higher type II fibre content. Also, there appears to be a limited effect of exercise training on Ca^2+^ sensitivity in type II fibres (Godard et al. 2002; Lynch et al. 1994; Malisoux et al. 2006), which would not improve their contractile capacity and thus not strongly influence RFD.

#### Interaction between neural and fibre-dependent mechanisms?

Both MU discharge rate and fibre type each appear to influence RFD, especially in the early rise in force. However, speculatively, the interactive (i.e., synergistic) effects of these factors might have a more potent influence on RFD for several reasons. First, high-threshold MUs that contain type II fibres generally have faster contraction speeds so their earlier recruitment as a result of increased MU discharge rates and lower recruitment thresholds ensure a faster whole-muscle force rise (Desmedt and Godaux 1978). Second, type II fibres have a greater Ca^2+^ release per action potential (Baylor and Hollingworth 2003), resulting from a 200 % greater ryanodine receptor content, larger number of junctional t-tubular segments (Franzini-Armstrong 2007) and greater total sarcoplasmic reticulum development (Luff and Atwood 1971; Schiaffino and Margreth 1969). Therefore, the Ca^2+^ release per action potential, and hence the increase in force per action potential, will be greater in type II fibres. Third, the rate of cross-bridge formation is Ca^2+^ dependent, being 3–8 times faster in type II than type I fibres and with the difference increasing with increases in Ca^2+^ concentration (Metzger and Moss 1990). Thus, the increases in Ca^2+^ release triggered by high discharge rates may have a cumulatively greater effect in type II fibres. Finally, type II fibres have a 200–300 % greater Na^+^ channel density, and therefore might be better able to conduct high rates of excitatory potentials resulting from high discharge rates (Schiaffino and Reggiani 2011). For these reasons, increases in neural drive to the muscle may have a more substantive influence on RFD in muscles that have a greater proportion of type II fibres. This theory is consistent with the rate of force rise early in a voluntary contraction being strongly related to both the rate of muscle activation as well as the rate of twitch force rise (as described above). This phenomenon was supported by the modelling results of Duchateau and Baudry (2014), who found that an increase in discharge rate up to 100–200 Hz augmented RFD of all MUs in a pool but that further increases in discharge rate preferentially influenced only the faster units (see “Association between maximal MU discharge rate and RFD”). A purpose of future research is to more explicitly examine the interactions between neural and muscular mechanisms that influence RFD to develop a more complex mechanistic model describing RFD change.

#### Muscle size and architecture

Maximal strength capacity is significantly correlated with voluntary RFD (Mirkov et al. 2004), and the strength of this relationship increases with time from contraction onset in a sigmoidal manner (Andersen and Aagaard 2006; Folland et al. 2014), e.g., MVC strength explained 18, 29, 57 and 78 % of the variance in voluntary RFD recorded over the first 10, 50, 100 and 200 ms, respectively, of a rapid voluntary contraction (Andersen and Aagaard 2006). It is therefore reasonable to suggest that factors influencing MVC strength (whose main determinants are neural drive and muscle cross-sectional area) may also influence RFD. Therefore, both the ability to activate the muscle at high force levels (which is not necessarily associated with the ability to rapidly activate the muscle at force onset) (de Ruiter et al. 2004) and muscle size might be influential (Andersen and Aagaard 2006; Erskine et al. 2014). The effect of muscle architecture on RFD is currently poorly understood, but increases in pennation (fascicle) angle allow for a greater muscle physiological cross-sectional area for a given muscle size (volume), and thus for a greater absolute rate of force rise (particularly later in the rise of force). Nonetheless, contractile forces are more directly transmitted to the tendon in muscles with lesser pennation; i.e., muscular force output decreases proportionally to the cosine of fibre angulation. During low-force isometric twitch contractions, where the effects of fibre length and rotation on force rise are minimal (due to little changes in fibre length or angle) (Spector et al. 1980), the net impulse (i.e., force–time integral) developed by a parallel-fibered muscle is greater than that of a pennate muscle (assuming identical peak forces are generated) (Spector et al. 1980). Nonetheless, the benefit of a parallel-fibred muscle design may not be so significant in some contexts as increases in pennation also increase fibre rotation during muscular contractions (i.e., gearing effects) (Azizi et al. 2008; Brainerd and Azizi 2005), which increases origin-to-insertion muscle shortening speed (Gans 1982) and possibly the rate of force rise. This is particularly useful in muscles that transfer forces through long tendons because substantial muscle shortening would occur as the tendon lengthens in the force transfer process. However, a direct investigation is needed to fully explore the impact of pennation angle on RFD in human skeletal muscle.

According to Edman and Josephson (2007), ~40 % of the variance in the force rise measured early in a contraction (to 50 % maximum force) is associated with the requirement to take up series elastic slack in muscle fibres, indicating that muscles with greater series compliance may have a slower RFD. Therefore, longer muscles, or muscles with longer fascicles, could theoretically exhibit a slower force rise because of a greater extent of series elastic material (i.e., actin–myosin filaments, titin protein, cross-bridges and potentially a need for a longer internal aponeurosis). Some preliminary evidence has been presented for this in human muscles (Blazevich et al. 2009) and it is well known that muscles developing high RFD during human movement, such as soleus and gastrocnemius during running and jumping, possess short muscle fibres (Lieber and Ward 2011). However, the plantar flexors have a slower isometric RFD than, for example, the knee extensors, probably because of the cost to rapid force rise of their pennate structure and because forces are delivered through a long and compliant structure (Achilles tendon and plantar fascia for plantar flexion) which slows the rate of force delivery (Wilkie 1949). Therefore, whilst muscle architectural characteristics likely influence the contractile properties of muscles, a lack of experimental data makes it difficult to accurately determine their true effect on RFD within a complex muscle–tendon system.

#### Musculotendinous stiffness

The speed of force transmission through a material is influenced by the material’s stiffness: $$v = \sqrt {{\raise0.5ex\hbox{$\scriptstyle {kx}$} \kern-0.1em/\kern-0.15em \lower0.25ex\hbox{$\scriptstyle \mu $}}}$$, where *v* is the force transmission velocity, *k* is the material’s stiffness, *x* is the length relative to resting length, and *μ* is the mass:length ratio of the material. As tissue stiffness is inversely proportional to length, longer tissues (both muscles and tendons) are more compliant and force transmission may be slower. This concept was clearly demonstrated by Wilkie (1949) and might explain the large differences between muscles; e.g., slower force transmission is expected for the plantar flexors, which work through the Achilles tendon and plantar fascia of the foot, than the quadriceps, which work through the shorter patellar tendon. It also may contribute to inter-individual differences in RFD. For example, the ~500 % variability in both patellar (Bojsen-Moller et al. 2005) and Achilles (Waugh et al. 2013) tendon stiffness was positively correlated with RFD obtained for respective agonist muscle groups (*r*^2^ = 0.29–0.90; measured to different time intervals). Nonetheless, changes in tendon stiffness induced by strength training (typically <50 %) (Wiesinger et al. 2015) are small when compared to the variability observed within a population (~500 %). A similar effect may be seen in detraining/unloading studies. For example, small reductions in vastus lateralis tendon stiffness (with “tendon” elongation measured at the fascicle–aponeurosis junction) after a period of bed rest were not correlated with the decline in RFD (*r*^2^ = 0.19, *n* = 6) (Kubo et al. 2000), suggesting that changes in tendon properties and RFD may be divergent. Since force transmission speeds through materials such as tendons are extremely fast (i.e., milliseconds for Achilles tendon) (DeWall et al. 2014; Nordez et al. 2009), considerably large changes in stiffness might be needed with training or disuse to significantly impact RFD.

Of note is the lack of information regarding the possible influence of muscle stiffness on RFD. If the rate of force transmission is influenced by tissue stiffness then changes in (active) muscular stiffness should theoretically impact RFD. Given the significant mass of muscle compared to tendon in humans, one might speculate that the effect of muscle stiffness on RFD may be more significant than that of tendon. Whilst no studies have explicitly isolated the effects of muscle stiffness on RFD, the influence of muscle–tendon unit stiffness on RFD has been examined. Hannah and Folland (2015) found muscle–tendon unit stiffness to be associated with late-phase RFD, although this effect appeared to be dependent upon maximum strength as relative RFD was unrelated to muscle–tendon unit stiffness. Theoretically, inter-individual and inter-muscular variations in RFD may be partly expected to be influenced by musculo-tendinous stiffness, however, existing data are inconsistent and a causative relationship has not been clearly demonstrated. Clearly, additional data are needed to evaluate these hypotheses.

### Adaptive changes in rapid force capacity with training

#### Contribution of neural factors

The ability to produce a rapid rise in contractile force during the initial phase of a voluntary contraction (0–300 ms), as reflected by a high RFD, is vital not only to the trained athlete but also to the elderly individual who needs to counteract sudden perturbations in postural balance. As discussed in this subsection, various modalities of strength training may evoke parallel increases in RFD and muscle activation (EMG amplitude, rate of EMG rise, H-reflex amplitude) during the initial phase of a voluntary contraction. Although these observations support the contention that neural adaptations are a strong contributor to the gain in RFD induced by training (Aagaard 2003), additional contributions may also occur from concurrent increases in muscle size, type II muscle fibre proportion and tendon stiffness characteristics.

**Fig. 3:**
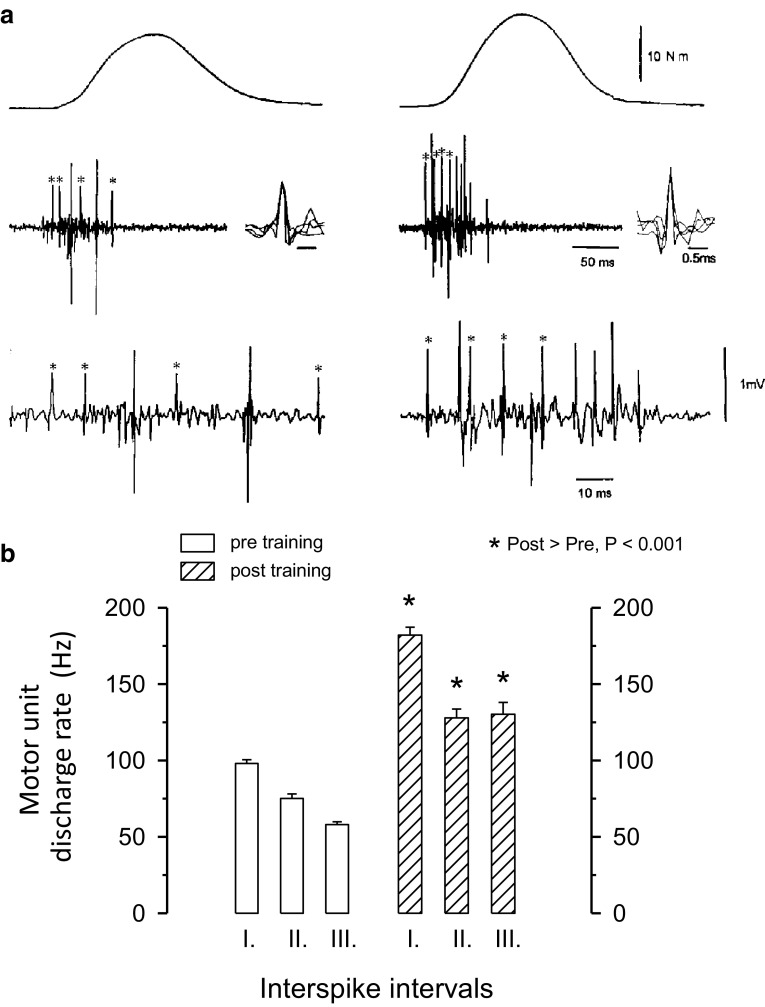
Motor unit (MU) discharge rate at the onset of maximal ballistic contractions, obtained in the tibialis anterior muscle before and after 12 weeks of ballistic-type strength training. **a** Representative MU action potential recordings obtained before (*left*) and after (*right*) training. Note the marked increases in MU discharge rate and RFD with training. **b** Mean discharge rate (and SEM) recorded in the first, second and third interspike intervals, respectively. All post-training values were greater than pre-training values (*p* < 0.001). The number of MU action potentials analysed in each interspike interval ranged between 243 and 609. Figure reproduced with permission from Van Cutsem et al. (1998) and Aagaard (2003)

**Fig. 4:**
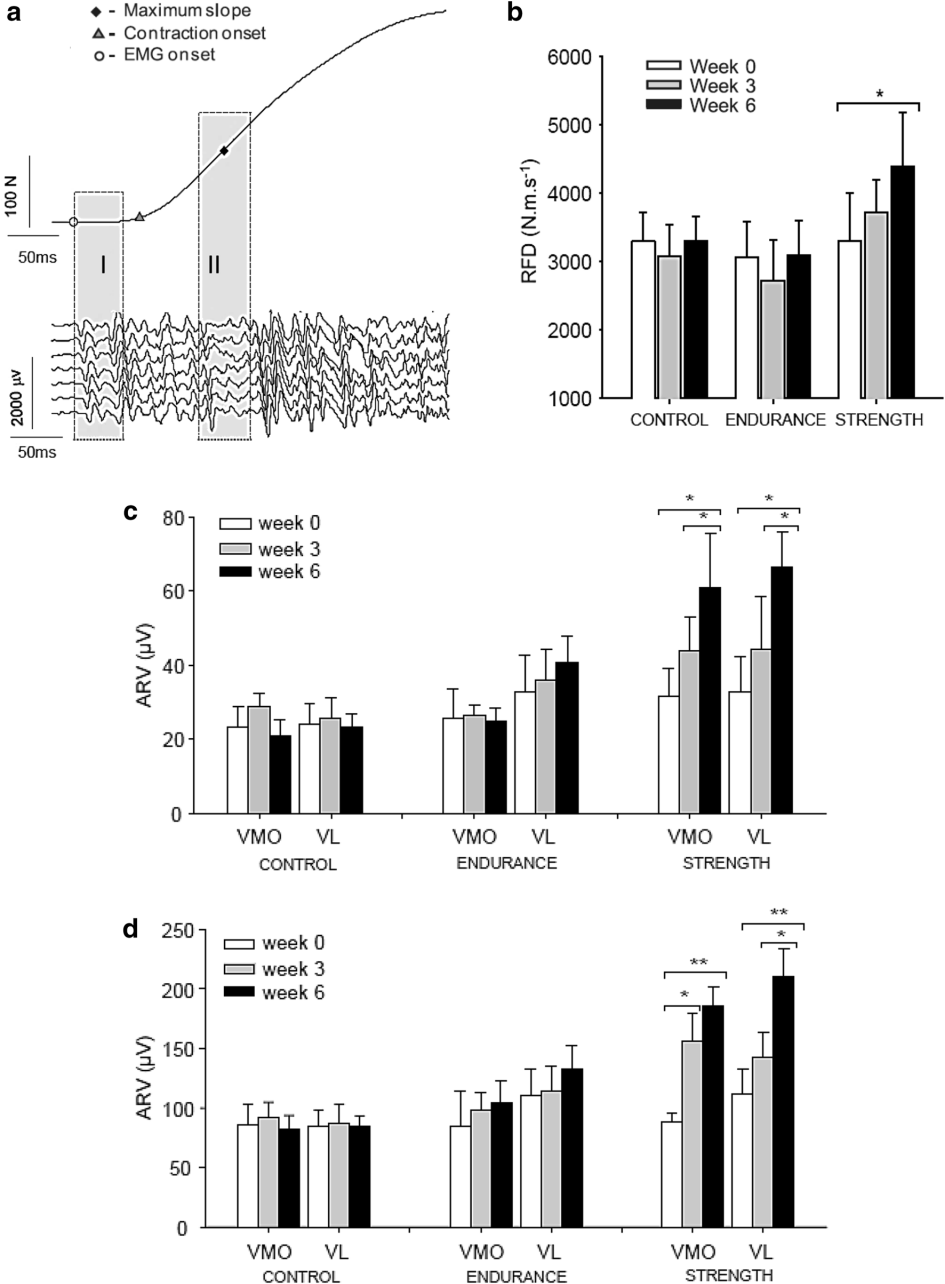
**a** Representative linear-array EMG recordings obtained in the vastus lateralis muscle during explosive isometric contractions of the knee extensors. EMG signals were analysed in two intervals of 50 ms (*shaded boxes* *I* and *II*) that were initiated 70 ms before the onset of force (*I*) and centered at the time instant of maximal RFD (peak slope) (*II*). **b** Mean RFD (and SEM) obtained before, during (3 weeks) and after 6 weeks of heavy-resistance strength training, endurance training or no training. *p < 0.05. **c**, **d** Average rectified EMG amplitude (ARV; mean and SE) measured for the vastus lateralis and vastus medialis muscles in time intervals *I* (**c**) and *II* (**d**). *p < 0.05; **p < 0.001. Note that increases in RFD and EMG activity were only observed following strength training, while absent in response to endurance training and in non-trained controls. Figure reproduced with permission from Vila-Cha et al. (2010)

Both heavy-resistance strength training and explosive-type strength training have a strong stimulatory effect on RFD. Driven by the strong influence of muscle activation on RFD demonstrated both in vitro and in vivo *(*see “Association between muscle activation and RFD”), parallel gains in RFD and muscle activation, the latter evaluated by EMG analysis, have been consistently observed following weeks to months of strength training (Aagaard et al. 2002a; Hakkinen et al. 1985, 2001a, 2003; Schmidtbleicher and Buehrle 1987; Tillin and Folland 2014) (for illustrative data obtained in a representative subject, see Fig. 3a). For example, 33 % higher RFD values were recorded in the knee extensors by Vila-Chã and co-workers following 6 weeks of lower-limb strength training (leg press, leg extension, and leg curl; 60–85 % 1-repetition maximum loads), which was accompanied by 80–100 % increases in EMG activity (Vila-Cha et al. 2010) (Fig. 4). Interestingly, no changes in RFD or muscle activation (EMG amplitude) were observed in response to 6 weeks of endurance training performed on a bicycle ergometer at an exercise intensity corresponding to 50–75 % heart rate reserve (Fig. 4).

Observations of a positive linear relationship between RFD and integrated EMG (de Ruiter et al. 2007; Del Balso and Cafarelli 2007; Klass et al. 2008) as well as between the training-induced increases in these parameters (Andersen et al. 2009; Blazevich et al. 2008) support that a causative relationship exists between the training-induced gains in muscle activation and RFD, respectively. Stressing that adaptive changes in neuronal motor function exert a strong influence on the gain in RFD induced by strength training, moderate-to-strong positive associations (*r*^2^ = 0.46–0.81) between training-induced changes in RFD and EMG amplitude (including rate of EMG rise) have been reported for the human quadriceps femoris (Blazevich et al. 2008; de Ruiter et al. 2012) and trapezius (Andersen et al. 2009) in response to 4–10 weeks of heavy-resistance strength training. As further evidence of a causal link between training-induced gains in muscle activation and RFD, systematic progressive improvements along the linear relationship between EMG activity and RFD were observed with successive training sessions during 4 weeks of isometric plantar flexor training (Del Balso and Cafarelli 2007).

In addition to potential cortical plasticity at the early stage of the learning process of a new motor task (see “Limiting mechanisms of MU discharge rate”), other training-related adaptations should occur at different levels of the neural system. Most likely, modulations in spinal circuitry contribute to the adaptation in RFD with strength training. In support of this notion, a positive correlation (*r*^2^ = 0.35) was observed between gains in RFD measured during MVC efforts and increases in soleus H-reflex amplitude recorded during isometric plantar flexions performed at submaximal force levels following 3 weeks of isometric plantar flexor training (Holtermann et al. 2007), the latter parameter suggesting improvements in spinal motor neurone excitability and/or Ia afferent synaptic transmission efficacy (Aagaard 2003). Addressing the aspect of spinal modifications with strength training and their importance for the adaptation in RFD, marked increases in the maximal discharge rate of MUs during rapid isometric dorsiflexions have been observed in response to ballistic-type strength training (Van Cutsem et al. 1998) and following more traditional heavy-resistance strength training regimes in both young and old adults (Christie and Kamen 2010; Kamen and Knight 2004; Patten et al. 2001). Given the governing influence of MU discharge rates on RFD (see “Association between maximal MU discharge rate and RFD”), the observation of very large increases in MU discharge rate at the onset of muscle contraction, along with an increased ability to sustain a high discharge rate from the first to the second and third interspike intervals, was suggested to be strongly responsible for the training-induced rise in RFD (Van Cutsem et al. 1998) (see Fig. 3). It is possible, however, that such large increases in MU discharge rate are most readily achieved by use of explosive-type exercise and/or ballistic movement training given the fact that discharge rates are 2–3 times higher during ballistic than slow contractions (Desmedt and Godaux 1977; Van Cutsem et al. 1998).

In addition to the increase in maximal MU discharge rates evoked by weeks or months of ballistic-type strength training in both young and old adults, an increased incidence of doublet discharges (i.e., successive MU action potentials with an interspike interval ≤5 ms) was observed following 12 weeks of dynamic ballistic training of the human dorsiflexors (tibialis anterior), representing a change towards ultra-high discharge rate behaviour (≥200 Hz) of MUs at the onset of ballistic muscle contractions (Van Cutsem et al. 1998). The presence of doublet discharges at the onset of muscle contraction is known to result in a marked increase in contractile force production, and in particular leads to large increases in RFD (Binder-Macleod and Kesar 2005; Burke et al. 1976; Moritani and Yoshitake 1998). Specifically, the incidence of such ultra-high discharge rates increased from 5 % of all MUs recorded prior to training to comprise 33 % of all MUs recorded following 12 weeks of ballistic strength training (Van Cutsem et al. 1998). This adaptive change in the discharge output of spinal motor neurones is likely to take increasing advantage of the so-called catch-like property of skeletal muscle, where the arrival of doublet action potentials at the motor end plate and their signalling throughout the cell membrane triggers an amplified magnitude of Ca^2+^ efflux from the sarcoplasmic reticulum to the cell cytosol (Duchateau and Hainaut 1986), in turn evoking an extraordinary steep rise in contractile force development (Binder-Macleod and Kesar 2005; Cheng et al. 2013).

Several lines of evidence suggest that exercise involving explosive-type muscle contractions (i.e., muscle actions performed with maximal intentional RFD) is the most efficient training modality, regardless of the training load used, for inducing maximal gains in RFD and muscle activation at contraction onset. For example, 4 weeks of isometric knee extensor training using a maximal intentional RFD and high peak force level (90 % MVC) produced markedly larger gains in RFD and muscle activation, respectively, than conventional hypertrophy training performed using lower intensities (75 % MVC) and submaximal RFD efforts (Tillin and Folland 2014). In addition, robust concurrent increases in RFD and EMG activity have been demonstrated by employing explosive-type resistance exercise in young adults (de Ruiter et al. 2012; Tillin and Folland 2014; Van Cutsem et al. 1998), old to very old individuals (Caserotti et al. 2008), and frail elderly patients recovering from elective hip replacement surgery (Suetta et al. 2004). These findings collectively indicate that explosive-type strength training is not only highly effective in eliciting marked gains in rapid force capacity (RFD and impulse) and increased muscle activation at the onset of muscle contraction, but is also tolerable across a wide range of individual backgrounds from young untrained/trained individuals to inactive frail elderly. On the other hand, it should be recognised that the use of non-explosive albeit heavy (≥75 % of 1-repetition maximum) training loads also seems effective in eliciting substantial increases in contractile RFD (Aagaard et al. 2002a; Andersen et al. 2009).

#### Contribution of musculotendinous factors

In addition to the adaptive changes induced in the nervous system in response to strength training described above (for more detailed reviews, see Aagaard 2003, 2010; Sale 2003), a number of contributing alterations might also be elicited in the musculoskeletal system (Aagaard and Thorstensson 2003; Folland and Williams 2007). Although beyond the main scope of this review, these contributing factors will be briefly discussed below to acknowledge that the training-induced adaptation of RFD during rapid contractions is not solely driven by changes isolated to the nervous system.

Duchateau and Hainaut (1984) were the first to demonstrate an important role of muscle adaptation in the adaptive gain in RFD induced by strength training. The authors reported 18–31 % increases in peak RFD values when electrical stimulation of the motor nerve was applied to evoke tetanic contractions of the human adductor pollicis following 3 months of explosive-type isometric or dynamic strength training. These observations strongly suggest that training-induced changes in RFD can be evoked independently (or even in the absence) of changes in muscle activation (i.e., adaptive changes in the central nervous system) (Duchateau and Hainaut 1984). Elaborating on the muscular component of RFD adaptation, increasing overall anatomical muscle size (cross-sectional area or volume) with training represents an effective means to increase RFD, since maximal contractile force capacity (and thereby RFD) is strongly governed by the macroscopic size of the muscle (Moss et al. 1997). Notably, increases in anatomical muscle cross-sectional area and/or volume appear to be a highly robust finding following heavy-resistance strength training in both young, old and very old adults (Aagaard et al. 2001; Esmarck et al. 2001; Hakkinen et al. 1998; Harridge et al. 1999; Holm et al. 2008; Narici et al. 1989; Suetta et al. 2004; Vikne et al. 2006).

While non-selective myofibre hypertrophy likely contributes to increased RFD by increasing MVC strength, preferential type II hypertrophy (Aagaard et al. 2001; Hakkinen et al. 2001b; Staron et al. 1990; Suetta et al. 2008) is expected to increase RFD to an even greater extent given the higher intrinsic RFD of type II versus type I fibres (Metzger and Moss 1990). Furthermore, given the 10–50 % higher specific force of type II compared to type I fibres observed in both young and old individuals (Bottinelli et al. 1999; Hvid et al. 2011; Larsson et al. 1997; Trappe et al. 2003), heavy-resistance exercise regimes evoking preferential type II hypertrophy are likely to achieve an even greater relative gain in MVC force and RFD compared to more non-selective training regimes. In support of this notion, Hakkinen et al. (1985) reported a positive association (*r*^2^ = 0.30) between the change in type II:I fibre ratio and the change in time to reach 30 % of MVC force after 24 weeks of explosive strength training. Further highlighting the influence of muscular changes on RFD, Andersen et al. (2010) found that a decrease in relative RFD (i.e., normalised to MVC) measured in the very initial contraction phase (0–50 ms relative to force onset) after 14 weeks of non-explosive heavy strength training was correlated with the decrease in the relative proportion of type IIX muscle fibres (*r*^2^ = 0.37).

It is well established that the content of myosin heavy chain IIX isoforms in human skeletal muscle is markedly reduced (50–90 %) along with a corresponding upregulation in myosin heavy chain IIA isoforms in response to prolonged heavy-resistance strength training protocols (Andersen and Aagaard 2000; Andersen et al. 2010; Kraemer et al. 1995). This interaction may account for the finding that short-term (e.g., 2–8 weeks) strength training programs tend to increase RFD significantly (e.g., Holtermann et al. 2007; Ogasawara et al. 2013; Tillin et al. 2012b), when increases in MU discharge rates are elicited without substantive fibre type transformation or hypertrophy, whereas longer periods of strength training may have less effect on (or might even reduce) RFD (Hakkinen et al. 1985; Ogasawara et al. 2013), when less change in MU discharge rate presumably occurs but reductions in type IIX myosin heavy chain content are triggered (Andersen et al. 2010). A purpose of future research, therefore, is to more explicitly examine the interactions between neural and muscular mechanisms that influence RFD to develop a more complex mechanistic model describing the adaptability in RFD with training.

Training-induced changes in muscle–tendon stiffness might speculatively (and partially) contribute to the observed increase in RFD with strength training. In support of this notion, it has previously been demonstrated that tendon and aponeurosis stiffness is positively related to RFD in vivo (Bojsen-Moller et al. 2005; Waugh et al. 2014) (see “Musculotendinous stiffness”) and that increased in-series compliance of the muscle–tendon unit leads to decreased RFD (Wilkie 1949). In addition, substantial increases (+15–25 %) in tendon stiffness have been reported for the patellar and Achilles tendons in response to prolonged periods of strength training in both young and old individuals (Arampatzis et al. 2007; Duclay et al. 2009; Kongsgaard et al. 2007; Reeves et al. 2003; Waugh et al. 2014; Seynnes et al. 2009). Thus, training-induced increases in tendon stiffness may potentially be a factor influencing RFD, at least partly, however, further research is required to confirm a causative role in this association.

#### Implications for selected subject groups

Cross-sectional studies show that top-level power athletes (track and field sprinters and jumpers) are characterised by a markedly greater knee extensor RFD measured during the initial 150 ms of muscle contraction than age-matched habitually active individuals, which may be partly explained by a greater muscle activation in power athletes at the initiation of muscle contraction (0–50 ms relative to force onset) (Tillin et al. 2010). When normalised to MVC, RFD represented by the force developed at specific time points was also greater in power athletes compared to non-athletes (Tillin et al. 2010), indicating the presence of qualitative adaptations in this group of athletes that could potentially rely on higher MU discharge rates and more pronounced doublet discharge behaviour, greater proportions of type II muscle fibres and/or elevated tendon stiffness.

The above observations, and their associated mechanisms in the neuromotor system, have important implications for athletic performance. For example, strength-trained athletes (elite rugby players) with a high RFD demonstrated substantially faster 5-m sprint (acceleration) times (<1 s) than athletes with a lower RFD (slower 5-m sprint times, ≥1 s) (Tillin et al. 2013a). Further underlining the importance of a high RFD for superior sprint/acceleration capacity in sports, a negative linear relationship (*r*^2^ = 0.42, *p* < 0.05) was observed between 5-m sprint time and relative RFD obtained at 100 ms (Tillin et al. 2013a).

The gains in muscle activation and particularly in maximal MU discharge rate induced by strength training, and the resulting improvement in RFD are of great importance not only for athletes but also for elderly individuals. In the case of untrained individuals, maximal MU discharge rates recorded during MVC efforts are reduced in old compared to young adults (Christie and Kamen 2010; Connelly et al. 1999; Kamen and Knight 2004; Klass et al. 2008). Moreover, maximal MU discharge rates are substantially reduced at the onset of rapid isometric muscle actions in old compared to young adults (Klass et al. 2008). As a consequence (although other factors such as type II myofibre atrophy contribute as well), maximal RFD is substantially reduced in old versus young adults (Barry et al. 2005; Clarkson et al. 1981; Hakkinen et al. 1998; Hvid et al. 2010; Izquierdo et al. 1999; Klass et al. 2008; Korhonen et al. 2006; Suetta et al. 2009). In turn, the age-related reduction in RFD and maximal MU discharge rate may present serious functional consequences for elderly people. In older individuals a reduced RFD is associated with a decreased postural stability during upright standing (Izquierdo et al. 1999) while a reduced rate of EMG increase, representing the neural equivalent of RFD, is associated with impaired balance recovery during tripping (Pijnappels et al. 2005).

Notably, life-long strength-trained elderly (weight lifters) demonstrate maximal MU discharge rates that are ~25 % higher than those observed in age-matched (67–79 years) untrained individuals (Leong et al. 1999), suggesting that the age-related decline in maximal MU discharge rates may, to a large extent, be counteracted by (particularly explosive) strength training. In strong support of this, maximal MU discharge rates recorded during MVCs appear to consistently increase in old as well as young individuals in response to strength training (Christie and Kamen 2010; Kamen and Knight 2004; Patten et al. 2001; Van Cutsem et al. 1998). Notably, when compared following a period of strength training, maximal MU discharge rates no longer seem to differ between old and young subjects (Christie and Kamen 2010; Kamen and Knight 2004; Patten et al. 2001), suggesting that heavy-resistance exercise is highly effective in removing the age-related deficit in maximal MU discharge rate during conditions of maximal muscle force production. This important adaptation in motor neurone function may explain the observation of marked gains in RFD in old (60 years) and very old (80 years) women when exposed to 12 weeks of explosive-type heavy-resistance strength training (Caserotti et al. 2008). Notably, the age-related deficit in RFD observed between the 60- and 80-year-old women was reduced from 43 % prior to training to 15 % following training with no statistical age-related difference observed (Caserotti et al. 2008).

### Concluding remarks

**Fig. 5:**
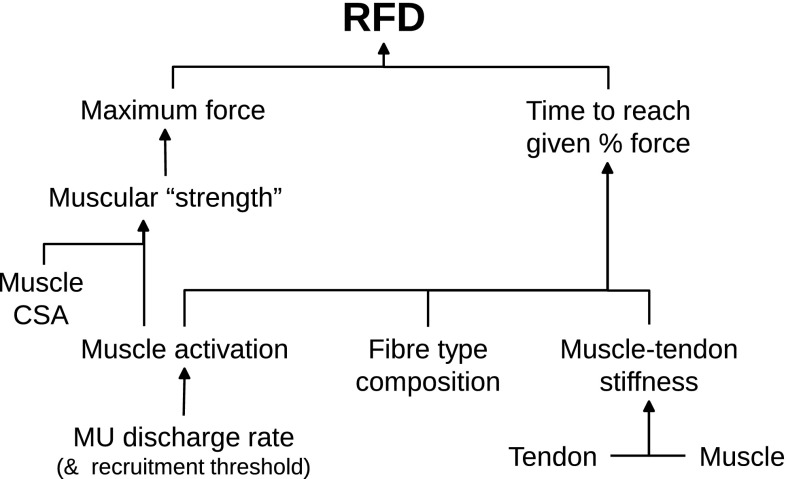
Rate of force development (RFD) is influenced by numerous factors within the neuromuscular system; those contributing to maximal muscular strength will improve the mean RFD (assuming the rate at which a given proportional force level is reached remains constant), whereas those affecting the time to reach a given force level may additionally influence the RFD measured at different time intervals during the rise in muscle force. *CSA* cross-sectional area

Increasing the maximal voluntary force-generating capacity by means of strength training may affect RFD by increasing it proportionally across the force–time curve (i.e., where time to attain a given relative force level, say 50 % MVC, remains unchanged). However, as shown diagrammatically in Fig. 5, this relative rate for force rise can be modulated by a number of factors. For example, rapid muscle activation through reductions in MU recruitment thresholds and (perhaps more significantly) increases in MU discharge rates at the onset of contraction appear to be major factors influencing RFD, particularly in the early (first 50–75 ms) rise in force during a maximal voluntary muscle contraction. The ability to activate the muscle rapidly may in turn be influenced by factors at both supraspinal (e.g., motor cortical outflow, agonist–antagonist control) and spinal (e.g., neuromodulatory synaptic input) levels. In addition, differences in muscle fibre type composition between muscles and between individuals may also influence the observed differences in RFD. Moreover, evidence exists to indicate both a synergistic and multiplicative relationship between the rate of muscle activation by the nervous system and fibre type composition, which may be explored more explicitly in future research. The effects of training-induced changes in muscle architecture (potentially of small-to-moderate effect) and tendon stiffness remain unclear, with changes in tendon mechanical properties potentially influencing the adaptive change in RFD with strength training, but more likely (and to a greater extent) influencing RFD variation between muscles and between individuals. Although the amount and rate of Ca^2+^ release from the sarcoplasmic reticulum likely influence the RFD in vivo, it is unclear whether training-induced alterations in muscle fibre Ca^2+^ sensitivity play a role.

To briefly summarise the observations and data presented in this section, it is clear that explosive-type strength training has a large positive effect on RFD, and that heavy-resistance strength training can also elicit improvements in rapid force capacity (particularly in the later rise in force during contraction). It is also clear that the effect of ageing (a reduced RFD is observed in older adults) as well as differences between athlete populations (faster RFD is observed in athletes with a speed-power training background) comply well with our understanding of the above factors in their influence of RFD in human skeletal muscle.

## Methodological considerations: RFD evaluation

Because RFD variables are typically measured in isometric conditions to control for the confounding influence of joint angle and angular velocity changes, this section will focus exclusively on isometric RFD, unless explicitly stated otherwise. It is notable that RFD measures are less reliable than MVC force, and substantially so during the early phase of contraction. Therefore, there is a need for a strict methodological approach to maximise reliability and to collect worthwhile data.

### Dynamometer and recording

#### Task

The purpose, whether practical (e.g., for performance/training monitoring, injury screening, fall or balance prediction) or experimental, largely determines the choice of task for RFD measurements. If the purpose is practical, the task for RFD measurement should be specific to the practical activity of interest, as RFD is known to be influenced by the muscle group(s) and joint angle(s) at which it is measured (Bellumori et al. 2011; de Ruiter et al. 2004; Tillin et al. 2012a). RFD has most often been measured in isolated single-joint tasks such as elbow flexion/extension (Barry et al. 2005; Bellumori et al. 2011; Sahaly et al. 2001), knee extension/flexion (Aagaard et al. 2002b; de Ruiter et al. 2004; Hannah et al. 2014; Tillin et al. 2010) and ankle plantar/dorsiflexion (Gruber et al. 2007; Van Cutsem and Duchateau 2005; Waugh et al. 2013), via the use of a commercial isokinetic dynamometer with a rotational torque transducer (Aagaard et al. 2002b; Waugh et al. 2013), or a custom-built dynamometer incorporating a linear strain gauge load cell measuring force (Hannah et al. 2014; Mirkov et al. 2004; Tillin et al. 2010). Measuring the individual’s external lever arm (e.g., from the linear strain gauge load cell to the joint centre) facilitates conversion between force and torque, and the use of torque values may facilitate wider comparison of data sets.

Occasionally, RFD has been measured during multiple-joint actions such as squats (Nuzzo et al. 2008; Tillin et al. 2013a), leg press (Hakkinen and Keskinen 1989; Marcora and Miller 2000), and mid-thigh pulls (Kawamori et al. 2006), typically via the use of a force plate (Tillin et al. 2013a) or a linear force transducer (Marcora and Miller 2000). Isolated single-joint tasks typically provide an experimentally controlled situation in which to assess the underlying physiological determinants of RFD, whereas multiple-joint tasks may be more appropriate/relevant for practical outcomes.

#### Compliance

**Fig. 6:**
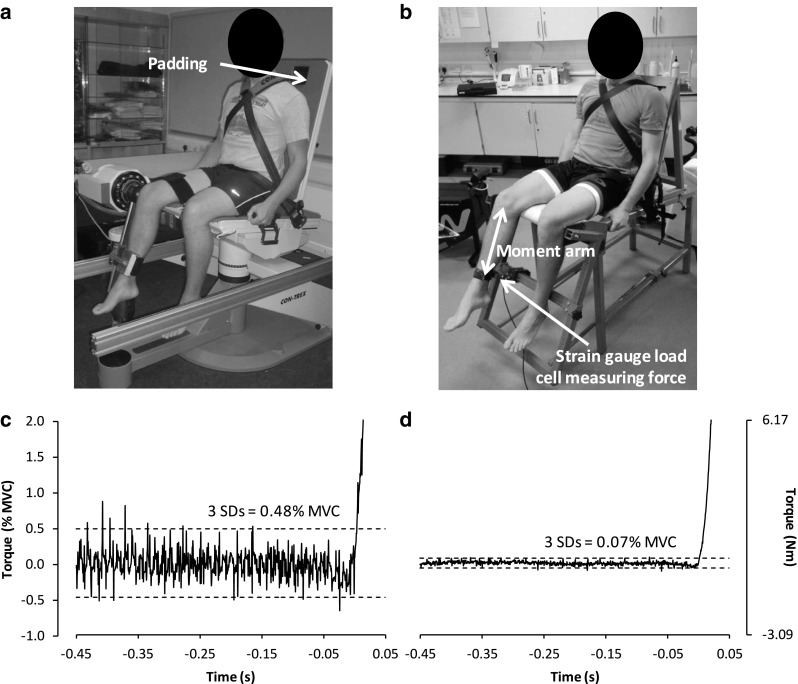
Photograph and baseline-torque recordings from a commercial isokinetic dynamometer (**a**, **c**) and a custom-built dynamometer (**b**, **d**), measuring knee extensor torque at ~60° knee flexion in the same participant. The padding on the chair/crank-arm adaptor in **a**, that is not present in **b**, contributes to dynamometer compliance. Torque in **d** is the product of force and moment arm. The baseline-torque recordings in **c** and **d** are prior to and during the early phase of an explosive contraction, and presented in the same scale for both absolute (Nm) and relative (% MVC) units. The dashed lines are ±3 SDs of the baseline mean which is often used as a threshold for detecting contraction onset, and is comparable with typical objective criteria thresholds for detecting an unstable baseline (e.g., shift above/below this threshold in the preceding 200 ms). The considerably greater baseline noise amplitude in **c** makes the thresholds for detecting contraction onset and/or an unstable baseline, including any pre-tension or countermovement, markedly less sensitive than in **d**

Any compliance or distensibility within the dynamometer system allows uncontrolled changes in joint angle and velocity (Wilkie 1949), as well as dissipation and thus attenuation of force during the period of movement. These effects confound RFD measurements and are highly undesirable. Some biological compliance is inevitable, however, due to compression of soft-tissue, leading to small unavoidable changes in joint angle. Nevertheless, compliance of the dynamometer depends on its mechanical rigidity and ability to restrain/minimise joint movement. Some dynamometers (particularly commercially available isokinetic dynamometers that are in large part designed for the comfort of patients; Fig. 6a) have been found to allow large changes in joint angle (>15° knee angle change during isometric knee extension) (Tsaopoulos et al. 2007) in comparison to rigid custom-built dynamometers (4° knee angle change during isometric knee extension) (Folland et al. 2014) (Fig. 6b), presumably due to the excessive compliance of commercial dynamometers. Compliance within the dynamometer is caused by non-rigid components, loose or poorly designed strapping and restraint of the participant, and excessive padding on the chair/table and crank-arm adaptor (Fig. 6a). We recommend using custom-built dynamometers or customising commercial dynamometers for minimal compliance.

For multiple-joint tasks (e.g., mid-thigh pull, squatting) there are more degrees of freedom and movement available within the musculoskeletal system which makes consistent positioning and restraint more problematic. In this instance it is advisable to start the explosive contraction from a standardized low-level of active pre-tension (Tillin et al. 2013a), even if it may partly affect peak RFD (Van Cutsem and Duchateau 2005).

#### Acquisition and filtering

The dynamometer used to measure RFD should ideally have low baseline noise amplitude, as this will improve the accuracy of the measurements and particularly the determination of contraction onset (discussed in more detail below). Commercial isokinetic dynamometers are often noisier than strain gauges (another reason for using custom-built dynamometers; see Fig. 6). For example, in knee extensor experiments baseline noise ranges of <0.1 % MVC force have been observed with custom-built dynamometers incorporating a strain gauge load cell (de Ruiter et al. 2006; Folland et al. 2014) compared with >1 % MVC force with a commercial isokinetic dynamometer (Tillin et al. 2012a).

Early-phase (first 25–50 ms) RFD appears important for performance (de Ruiter et al. 2006; Tillin et al. 2010) and injury avoidance (Domire et al. 2011; Krosshaug et al. 2007), yet can be small, e.g., 2–12 % of MVC force (Tillin et al. 2012a). Therefore it is important that the recording apparatus (strain gauge load cell, amplifier, sampling hardware and software) has low-noise and high-amplitude resolution to discern differences in early-phase RFD between individuals or sessions. To give an example, recent studies have used apparatus that allow force measurements to be resolved to 0.2 N over a 1 kN range (1 part in 5000) (Haider and Folland 2014).

The force signal should be sampled at a high frequency (≥1 kHz; ≥1 data point per millisecond) for several reasons: (1) to accurately measure the high RFD that human skeletal muscle is capable of producing (>10 maximum isometric forces per second) (de Ruiter et al. 1999); (2) to accurately identify contraction onset (Tillin et al. 2013b); (3) to synchronise the force signal with EMG, which has a Nyquist limit of ≥1 kHz (Konrad 2006); (4) to accurately measure motor response times such as electromechanical delay, which can be <7 ms for involuntary and <13 ms for voluntary contractions (Tillin et al. 2010); and (5) because the high-frequency (low-amplitude) baseline noise can provide a useful signal pattern from which contraction onset is manually identified (Tillin et al. 2013b). Following data acquisition there should be minimal filtering or smoothing of the signal to maintain the baseline noise pattern (if manually identifying contraction onset) and to avoid time shifts in the force signal caused by the smoothing function, which are particularly problematic if relating contraction onset to the onset of other biological responses, e.g., EMG to determine electromechanical delay. If filtering is unavoidable due to high baseline noise amplitude then we recommend using a zero lag, low-pass digital filter (e.g., fourth-order Butterworth) (Winter 1990) at the highest possible cut-off frequency, to minimise time shifts.

### Protocol

#### Instruction and feedback

RFD measurements are sensitive to the instructions given to the participant prior to the contraction. In an early study, Bemben et al. (1990) compared the force–time characteristics of isometric handgrip contractions in response to two different instructions: (1) you should reach peak force as quickly as possible by squeezing “hard and fast” versus (2) peak force is of no concern, and you should just squeeze as “fast” as possible. Whilst peak forces were greatest in the “hard and fast” condition, peak RFD was greatest in the “fast” condition. Very similar results were observed during both isometric elbow flexion and leg press exercises by Sahaly et al. (2001), who reported 20–46 % improvements in peak RFD when participants were instructed to push “fast” compared with “hard and fast” (see also Duchateau and Baudry 2014). Collectively, these results highlight that instruction should emphasise the importance of contracting as “fast” as possible if the aim is to achieve maximal RFD, and attempting to achieve maximal force and RFD within the same contraction may result in suboptimal measures of both parameters. Thus, contractions used to measure MVC force should be separated from those used to measure RFD (whenever possible), and the instruction in both situations should be specific to the objective of that contraction, i.e., as “hard” as possible for MVC force or as “fast” as possible for RFD. However, RFD has been found to have a strong positive relationship with the peak force achieved in that contraction (Bellumori et al. 2011; Van Cutsem et al. 1998). This suggests that participants should be encouraged to achieve high peak forces even if the emphasis is on contracting as fast as possible to measure RFD. Therefore, we recommend instructing participants to contract as “fast and hard” as possible with the emphasis on the “fast/explosive/rising” phase of contraction, and discarding any contractions with low peak forces, e.g., <70 % (de Ruiter et al. 2004) or <80 % MVC force (Folland et al. 2014).

If using distinct contractions to measure RFD (i.e., separate from those used to measure MVC force), we recommend keeping the explosive (“fast and hard”) contractions short (0.5–1.5 s) (Barry et al. 2005; Bellumori et al. 2011; Tillin et al. 2010; Van Cutsem et al. 1998). This minimises any fatigue or discomfort, which may be favourable in clinical patients, and enables completion of a larger number of trials (≥10) in a short period of time (e.g., 15–20-s rest periods between efforts) which might be expected to facilitate reliable and representative measures of RFD (see below).

During a test session it is important to provide visual RFD feedback to the participant after each explosive contraction, with appropriate explanation/interpretation, and encouragement to improve performance. Examples from the literature of how this feedback has been provided include displaying peak RFD (Tillin et al. 2010) or the time between 2–30 % of MVC force (de Ruiter et al. 2004). Within the RFD literature investigators have typically imposed the start of an explosive contraction through an auditory signal (e.g., 3-2-1-go); however, it is unknown whether this results in greater RFD measures than if the participant self-selects the start of the contraction.

#### Pre-tension and countermovement

Active tension in the muscle prior to the onset of an explosive contraction (pre-tension) alters the shape of the rising force–time curve increasing the initial (40 ms) torque-time integral (de Ruiter et al. 2006) and decreasing peak RFD (Van Cutsem and Duchateau 2005), in part due to a change in MU discharge pattern during the explosive action (Van Cutsem and Duchateau 2005) (see “Association between maximal MU discharge rate and RFD”). Similarly, a countermovement (i.e., the production of a negative/antagonist force) immediately prior to the onset of an explosive contraction also appears to influence RFD, as a function of the amplitude and duration of the countermovement (Grabiner 1994; Kamimura et al. 2009). Therefore, pre-contraction conditions should be standardised across contractions, participants and sessions to ensure reliable measures of RFD, and contractions with uncontrolled pre-tension or countermovement should be rejected. On a practical level, standardising the pre-contraction conditions can be done by displaying the baseline force on a sensitive scale in real time during the test session (Tillin et al. 2010) to provide this feedback to participants, and rejecting attempts where the baseline force is not sufficiently stable (e.g., shift >0.5 N in the preceding 200 ms) (Blazevich et al. 2009; de Ruiter et al. 2004; Tillin et al. 2010). During off-line analysis, objective criteria for a stable baseline should also be strictly applied. It is worth noting that the magnitude of countermovement/pre-tension that can be detected during the test session or analysis is dependent on the baseline noise, with lower noise amplitude facilitating a more sensitive detection of countermovement/pre-tension (Fig. 6).

#### Familiarisation and number of trials

Due to the need to maximise the reliability of RFD measures, we would recommend participants to complete a minimum of one familiarisation session prior to any measurements. Whilst there is no quantitative data describing the influence of the number of contractions on reliability, we recommend that investigators ensure they have collected at least five acceptable trials (stable baseline, high RFD score, explosive contractions) and average across the best three efforts to provide maximal RFD measures. If there is a clear trend toward an increase or decrease of RFD variables across the different trials, then this may signify potentiation or fatigue effects. Prolonged warm-up/practice procedures and/or inter-trial rest periods may potentially alleviate the problem.

### Analysis

As discussed above, contractions with discernible countermovement or pre-tension (according to objective criteria) should be excluded from the analysis. The majority of measurements available from the rising force–time curve involve determination of the onset, or start, of the contraction.

#### Contraction onset

Different onset detection methods have been proposed including threshold methods and systematic manual/visual approaches. Within the wider scientific literature on detecting signal onsets manual/visual approaches are generally considered as the gold standard/reference for validation of other approaches, i.e., automated detection methods (Di Fabio 1987; Soda et al. 2010; Uliam Kuriki et al. 2011). Within the RFD literature, determining contraction onset as the point at which force increases above a specified threshold has been widely employed, and as identifying this point is easily automated the method is considered reliable and time efficient. The threshold level has been set in absolute units (e.g., 7.5 Nm) (Andersen and Aagaard 2006; Blazevich et al. 2009) or relative to individual MVC force [e.g., 2 % MVC (Granacher et al. 2009) or 2.5 % MVC (Johnson et al. 2015; Rousanoglou et al. 2010)]. Whilst absolute thresholds provide a simple approach they may be unsuitable for comparisons of individuals, cohorts or muscle groups that have differing levels of function. Therefore, relative thresholds would seem to be preferable assuming they are based on a sufficiently robust reference measure.

**Fig. 7:**
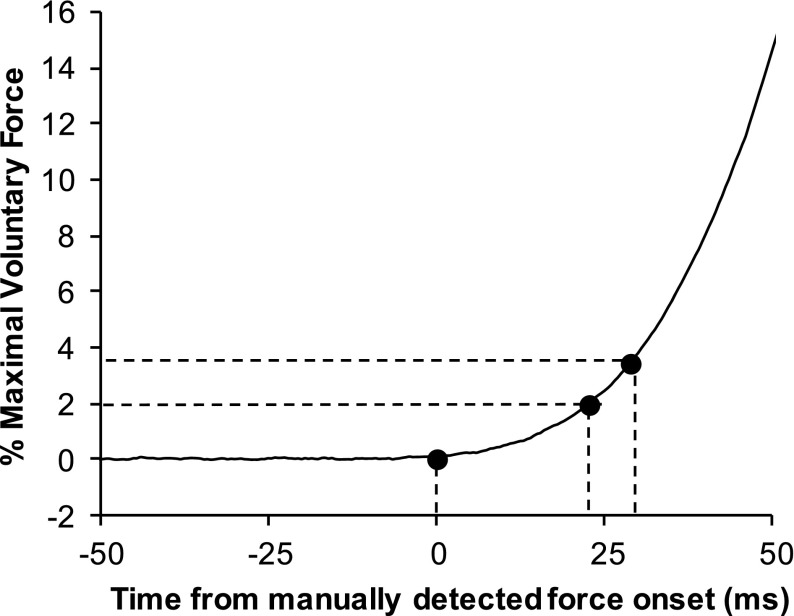
An unfiltered force–time curve recorded during an explosive contraction of the knee extensors (force is expressed relative to maximal voluntary force). Force onset (0 ms) was detected manually/visually using the systematic method detailed in Tillin et al. (2010). Some automated systems for detecting contraction onset have used arbitrary thresholds between 2 and 3.6 % maximal voluntary force which in this example occurs 24–30 ms after manually detected onset. Figure reproduced with permission from Tillin et al. (2013b)

As can be seen from the values above, studies utilising automated threshold methods (including absolute and relative) have often employed relatively high thresholds, likely due to the use of commercially available dynamometers that tend to have high inherent noise (noise range of ~5 Nm or >1 % MVC; Fig. 6c) (Tillin et al. 2012a) in comparison to custom-built dynamometers (<0.1 Nm; Fig. 6d) (de Ruiter et al. 2007; Tillin et al. 2010). These high thresholds may be relatively inaccurate in defining force onset, as recent studies using a low-noise dynamometer and systematic manual onset detection have found that knee extensor torques of >5 Nm or 2.5 % MVC are not achieved until >25 ms after contraction onset (Haider and Folland 2014; Hannah et al. 2012) (Fig. 7). This degree of inaccuracy for high-threshold automatic methods may invalidate measurements of RFD during the early phase of the contraction (e.g., first 50 ms). An alternative method for defining an automatic threshold is relative to the noise of the baseline force recording (e.g., 3 SDs; Fig. 6), which has produced low thresholds when used with custom-built dynamometers (~1 N or 0.5 Nm) (de Ruiter et al. 2004, 2006).

A wholly different approach involves systematic manual/visual identification of the actual instant of force onset. Whilst this approach appears to provide good validity (Tillin et al. 2013b), the potential influence of subjectivity could compromise its reliability (Staude and Wolf 1999), and it is undoubtedly less time efficient than automated methods. To maximise reliability of manual onset detection, these methods employ a systematic approach that includes the use of specific definitions to determine the actual instant of onset, e.g., “the last trough before force deflects above the range of the baseline noise” (Folland et al. 2014; Tillin et al. 2010) or “the first derivative of the filtered torque signal crossed zero for the last time before torque rise” (de Ruiter et al. 2007), and viewing the force recordings on a consistent scale (e.g., 300 ms vs. 1 N). When utilised with a low-noise dynamometer and minimal filtering this approach has been found to produce reliable onsets (intra- and inter-investigator SD of 0.33 and 0.52 ms) (Folland et al. 2014) and this inconsistency appears small in comparison to the >25 ms delay in onset identification with some high-threshold automated methods. However, with a high-noise dynamometer or extensive filtering/smoothing the manual visual method may be substantially less reliable.

In summary, if the initial phase of the rising force–time curve is of interest then a low-threshold automated method or a systematic method of manual onset detection is recommended. In either case, accurate onset detection is largely dependent on the noise of the dynamometer.

#### Variables

**Fig. 8:**
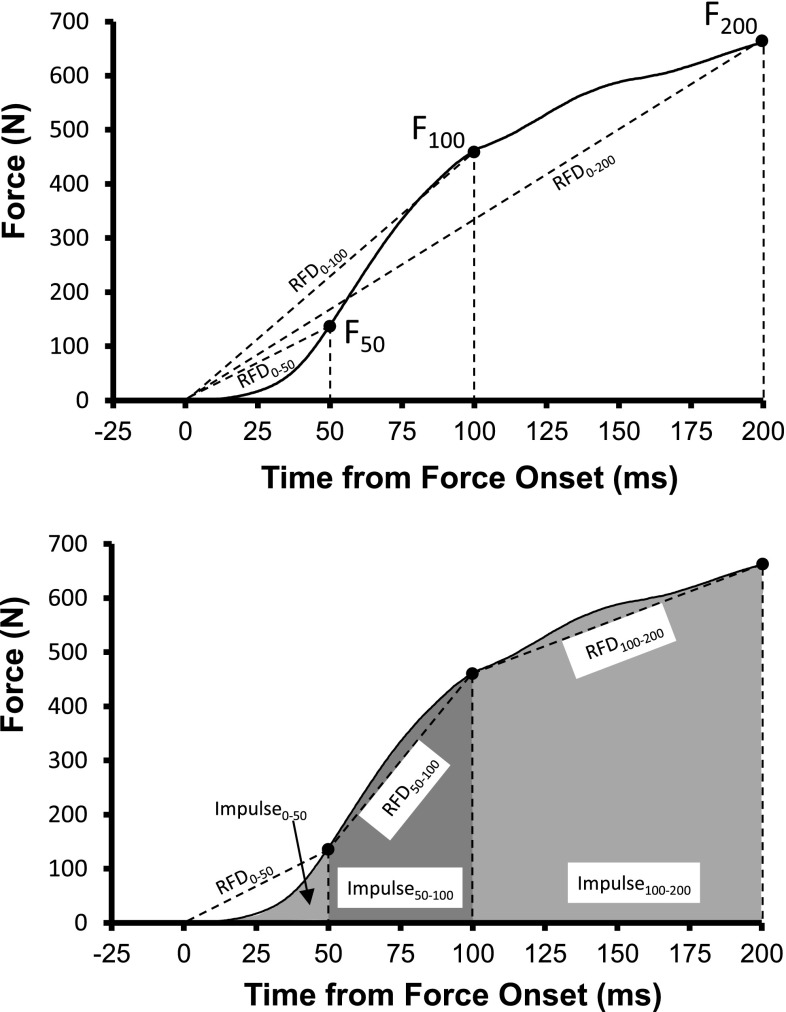
Common measurements of the rising force–time curve. **a** Force at specific time points (*F*_50_, *F*_100_, etc.) and overlapping RFD measurements all starting from force onset (RFD_0–50_, RFD_0–100_, etc.). **b** An identical force trace showing measurements of sequential RFD and sequential impulse both assessed over consecutive periods

Once contraction onset has been defined, a number of different measures can be obtained from the rising force–time curve. Some of the more common include force at specific time points (Rousanoglou et al. 2010; Tillin et al. 2010) (Fig. 8a) as well as RFD and impulse, which can both be measured over either overlapping periods starting from 0, e.g., RFD/impulse from 0 to 100 ms relative to onset (Aagaard et al. 2002b; Barry et al. 2005) (Fig. 8a), or consecutive periods, e.g., RFD/impulse from 50 to 100 ms relative to onset (Folland et al. 2014; Penailillo et al. 2015) (Fig. 8b). Irrespective of the measure, in all cases multiple time points/periods clearly provide a more comprehensive description of the whole rising curve than single measures. In addition, all measures should be corrected for the gravitational force on the limb.

Force at a specific time point from 0 (e.g., 300 N at 100 ms) is numerically equivalent to RFD from 0 to that point (3 N/ms), so it is strongly recommended not to report both of these measures to avoid redundant data. Consecutive RFD measurements provide additional information that may help identify when any divergence in the force–time curve occurs and isolate the physiological determinants contributing to this divergence (Folland et al. 2014). Another widely used variant of RFD is peak RFD, i.e., the steepest part of the curve over a specific epoch. Smaller epochs will be more sensitive to changes in the slope of the curve, but also more sensitive to unsystematic variability and thus less reliable, so thought should be given to the purpose of the measure (e.g., relevance to a functional action or physiological mechanism) before selecting an epoch. Nevertheless, peak RFD is a single metric at an inconsistent point on the force–time curve—except for brief impulse-like ballistic contractions (Bellumori et al. 2011; Casartelli et al. 2014; Klass et al. 2008; Van Cutsem et al. 1998)—and thus provides a less comprehensive and standardised measure than RFD or impulse over set time periods.

During dynamic functional activities, the change in momentum of a body/limb is directly proportional to impulse, and thus impulse is arguably the most functionally relevant RFD measure that can be taken during the rising force–time curve of explosive isometric contractions. It is therefore surprising that this measure has not been more widely used.

Another less common approach is to use time as the independent variable and assess time to achieve a specific force (Hakkinen and Keskinen 1989; Hannah and Folland 2015), or the time to increase from one force to another (Mirkov et al. 2004). Using time as the independent variable may be relevant to tasks where a specific force must be achieved. This measurement also facilitates comparisons over the same range of forces and may relate more directly to the physiological determinants specific to that range of forces (which may not be the case for fixed time periods with achievement of variable forces that involve changing determinants). Of the various measures discussed, time between two different forces, and peak RFD are the only measures that are independent of contraction onset.

#### Normalisation

Normalisation of the measures taken from the rising force–time curve can provide additional information. Normalisation to body mass may be useful for comparing individuals or groups of differing body size. The ideal normalisation function (i.e., ratio or allometric scaling) is beyond the scope of this review but may depend on the kinetic variable assessed (force or torque) (for more information see Folland et al. 2008; Jaric 2002). Another common normalisation procedure is comparison of RFD variables to MVC force or torque, which measures the ability to express the available force-generating capacity in an explosive situation. A less common, but physiologically revealing normalisation procedure is to compare voluntary RFD to equivalent values from an evoked contraction (de Ruiter et al. 2004; Tillin et al. 2012b). Specifically, an evoked octet of 8 supramaximal pulses at 300 Hz has been shown to drive the muscle at its maximal capacity for RFD during the early phase of contraction (de Ruiter et al. 2004; Deutekom et al. 2000). The voluntary:octet force ratio at 50 ms into the contraction has been termed “neural efficacy” (Buckthorpe et al. 2012; Hannah et al. 2012) as it indicates the effectiveness of volitional neural drive to utilise the muscle’s full capacity for explosive strength/RFD.

#### Reliability

Based on simple test–retest correlation analyses, high-to-moderate reliability has been documented for the assessment of RFD in the knee extensors (Buckthorpe et al. 2012), elbow flexors (Mirkov et al. 2004) and plantar flexors (Clark et al. 2007) as well as in isometric multi-joint movements such as the static squat (Tillin et al. 2013a). Despite the use of strict methodological procedures, RFD variables have been found to be less reliable than MVC strength (within-subject CV of 2–4 %) (Tillin et al. 2010, 2011, 2013a). In particular, the reliability of RFD and impulse measurements has consistently been found to be lower during the early phase of contraction, e.g., within-subject CV for force measurements over repeated measurement sessions: 12.8–16.6 % (0–50 ms); 4.5–5.3 % (0–100 ms); 4.5–5.1 % (0–150 ms) (Buckthorpe et al. 2012; Tillin et al. 2011), and other studies have found notably lower reliability (Jenkins et al. 2014a). Regarding consecutive RFD measures during the different phases of an explosive contraction, RFD from 50 to 100 ms relative to onset has been found to be more reliable than RFD from 0 to 50 ms or RFD from 100 to 150 ms relative to force onset, but similar to peak RFD, which is not surprising as peak RFD typically occurs within this middle time period (Buckthorpe et al. 2012). Impulse measurements have been found to have similar reliability to RFD measurements over the same periods (Buckthorpe et al. 2012), and qualitatively we have observed that there is often agreement between the two measures.

The high variability in early-phase volitional RFD measures appears likely to be due to neural factors, as measurements of contractile explosive force at the same time point are markedly less variable (supramaximal evoked octet CV 50 ms: 3.3–5.4 %) (Buckthorpe et al. 2012; Folland et al. unpublished data). The evoked octet has also been found to be a more reliable measure of evoked explosive force than the contractile response to a single twitch (Buckthorpe et al. 2012).

### Additional remarks

Whilst this section focuses on the assessment of isometric RFD, it is important to give some consideration to RFD obtained during dynamic contractions, as logically these are more relevant to functional tasks. In dynamic isoinertial contractions the mechanics of the system (joint angle, velocity and acceleration) interact with force/torque in a non-linear manner—according to the torque–angle-velocity relationship (King et al. 2006) and Newton’s second law of motion—confounding the evaluation of RFD. Nevertheless, it is conceivable that RFD and its determinants may be influenced by contraction type. Tillin et al. (2012a) compared torque at 25-ms time points from torque onset in concentric, eccentric, and isometric explosive knee extensions. The concentric and eccentric contractions were performed at a constant acceleration (2000° s^−2^), and torque at each time point in each condition was normalised to the MVC torque at that specific joint angle and velocity, to control for the confounding influence of angle and velocity changes. This normalisation approach also enabled comparison of the ability to utilise the available MVC torque in the different conditions. Results showed that normalised RFD was 60 % greater in concentric compared to eccentric and isometric conditions at all measured time points, and that available MVC torque could be achieved in less than half the time (≤125 ms) in concentric contractions than previously recorded in isometric contractions (>300 ms) (Aagaard et al. 2002a; Thorstensson et al. 1976). This appeared to be attributable to neural mechanisms as the authors also measured a greater voluntary:evoked torque ratio and EMG amplitude during the concentric condition. More recent work (Tillin et al. 2013c) has shown that the influence of contraction type on RFD is further delineated by contractile velocity, whereby the ability to utilise the available MVC torque in explosive actions is greatest in fast concentric contractions, followed by slow concentric, isometric, and eccentric contractions. Collectively, these studies provide unique evidence that dynamic contractions influence the ability to develop a high RFD, although more work is required to understand this phenomenon and its relevance to function.

### Concluding remarks and recommendations

To briefly summarise the observations presented in this methodological section, we recommend the application of rigorous procedures for the assessment of RFD, which include the following precautions:

1. using rigid custom-built dynamometers (or customising commercially available dynamometers) where possible to minimise both compliance and baseline noise;
2. sampling the force signal at more than 1 kHz to maximise accuracy;
3. avoiding (or minimising) signal filtering and smoothing to maintain baseline noise and prevent time shifts;
4. completing a separate familiarisation session;
5. instructing participants to contract “as fast and hard as possible” with particular emphasis on a fast increase in force;
6. using short (~1 s) contractions interspersed by short rest periods (e.g., 20 s) to record RFD separately from MVC force, where possible;
7. collecting at least five good contractions, from which the average RFD of the three best trials is retained;
8. rejecting trials with an unstable baseline (uncontrolled pre-tension and visible countermovement);
9. detecting the force onset with a low-threshold automated or systematic manual methods;
10. quantifying RFD/impulse at multiple time points rather than at a single instant;
11. considering that reliability is consistently lower during the early phase of the contraction.

## Conclusion

For more than five decades, isometric MVC strength has been extensively quantified to characterise in vivo skeletal muscle function in a variety of subject populations and conditions. The widespread use of this variable has resulted from its relatively easy (and valid) evaluation in both laboratory and clinical settings as well as to its good sensitivity to detect acute and chronic changes in neuromuscular function, while its functional value has often been questioned (see, e.g., Aalund et al. 2013). Since their first evaluation back in the 1970s (Thorstensson et al. 1976), RFD outcomes have been considered to have important functional consequences because of their temporal similarity with respect to sport and daily activities (Aagaard et al. 2002a) and because of their positive correlation with performances in both sporting and functional daily tasks (see, e.g., Maffiuletti et al. 2010; Tillin et al. 2013a). Contrary to single-measure MVC strength, there are multiple RFD variables that are differently governed by neural and muscular determinants depending on their temporal occurrence and quantification. One of the key points of this narrative review is that rapid muscle activation—likely through the critical role of MU discharge rate—may be considered as the main determinant of RFD in the early phase of the contraction (first 50–75 ms). Thus, changes in early-phase RFD estimates associated with fatigue, training or detraining have the potential to be used as a surrogate for neural function when techniques such as EMG and twitch interpolation are not implementable, e.g., for the evaluation of specific muscle groups (such as those around the hip) (Casartelli et al. 2014), in the applied/clinical setting. Another important point developed in our present article deals with the effectiveness of explosive-type and heavy-resistance strength training to improve the earlier (through an improvement in rapid muscle activation) and later rise in force, respectively, which have crucial implications for designing successful training and rehabilitation programs for athletes, elderly individuals and patients with different pathologies. A final key point of this current review is that the evaluation of RFD is quite challenging from a methodological point of view (much more than the simple assessment of pure MVC strength), primarily because of the large variability in rapid muscle activation capacity at the onset of the contraction, and therefore it should be conducted with particular care. The recommendations we have provided should favour a more valid and widespread quantification of RFD—as a complement, or alternative, to MVC strength—in both laboratory and clinical settings.

## Abbreviations

EMG: Electromyogram
MU: Motor unit
MVC: Maximal voluntary contraction
RFD: Rate of force development
